# Patient outcomes by baseline pathogen resistance phenotype and genotype in CERTAIN-1, a Phase 3 study of cefepime-taniborbactam versus meropenem in adults with complicated urinary tract infection

**DOI:** 10.1128/aac.00236-24

**Published:** 2024-05-23

**Authors:** Greg Moeck, Leanne B. Gasink, Rodrigo E. Mendes, Leah N. Woosley, MaryBeth Dorr, Hongzi Chen, Florian M. Wagenlehner, Tim Henkel, Paul C. McGovern

**Affiliations:** 1 Venatorx Pharmaceuticals, Inc., Malvern, Pennsylvania, USA; 2 LBG Consulting, LLC, Saint Davids, Pennsylvania, USA; 3 JMI Laboratories, North Liberty, Iowa, USA; 4 Justus Liebig University, Giessen, Germany; Tufts University - New England Medical Center, Boston, Massachusetts, USA

**Keywords:** cefepime-taniborbactam, β-lactamase inhibitor, meropenem, carbapenemase, outcome, complicated urinary tract infection

## Abstract

CERTAIN-1 was a Phase 3, double-blind, randomized, parallel group study of the efficacy and safety of cefepime-taniborbactam versus meropenem in the treatment of adults with complicated urinary tract infection (cUTI), including acute pyelonephritis. We determined susceptibility of Enterobacterales and *Pseudomonas aeruginosa* baseline pathogens to cefepime-taniborbactam and comparators and characterized β-lactam resistance mechanisms. Microbiologic response and clinical response were assessed in patient subsets defined by baseline pathogens that were of cefepime-, multidrug-, or carbapenem-resistant phenotype or that carried β-lactamase genes. Among Enterobacterales baseline pathogens, 26.8%, 4.1%, and 3.0% carried genes for extended-spectrum β-lactamases (ESBLs), AmpC, and carbapenemases, respectively. Within each treatment group, while composite success rates at Test of Cure in resistant subsets by pathogen species were similar to those by pathogen overall, composite success rates in meropenem patients were numerically lower for cefepime-resistant *Escherichia coli* (9/19; 47.4%) and ESBL *E. coli* (13/25; 52.0%) compared with *E. coli* overall (62/100; 62.0%). Cefepime-taniborbactam achieved composite success in 7/8 (87.5%) patients with carbapenem-resistant Enterobacterales and 8/9 (88.9%) patients with Enterobacterales with a carbapenemase gene (5 OXA-48-group; 2 KPC-3; 2 NDM-1). Cefepime-taniborbactam also achieved composite success in 8/16 (50.0%) patients and clinical success in 13/16 (81.3%) patients with *P. aeruginosa*; corresponding rates were 4/7 (57.1%) and 6/7 (85.7%) for meropenem. Cefepime-taniborbactam demonstrated efficacy in adult cUTI patients with cefepime-, multidrug-, and carbapenem-resistant pathogens including pathogens with ESBL, AmpC, and carbapenemase genes.

**CLINICAL TRIALS:**

This study is registered with ClinicalTrials.gov as NCT03840148.

## INTRODUCTION

Widespread use of antibiotics including carbapenems has resulted in the global dissemination of public health threat pathogens including extended-spectrum β-lactamase (ESBL)-producing Enterobacterales, carbapenem-resistant Enterobacterales (CRE), carbapenem-resistant *Pseudomonas aeruginosa* (CRPA), and multidrug-resistant (MDR) *P. aeruginosa* (1, 2). Strains of CRE and CRPA producing metallo-β-lactamases are emerging (3–6), with few treatment options available (7).

Taniborbactam is an investigational β-lactamase inhibitor that restores cefepime activity against cefepime-, carbapenem-, and MDR Enterobacterales and *P. aeruginosa* producing ESBLs, AmpC enzymes, serine carbapenemases, and NDM and VIM metallo-β-lactamases (8–12). In the Phase 3 CERTAIN-1 cUTI study (13), cefepime-taniborbactam was superior to meropenem for the primary composite endpoint (microbiologic and clinical success) at Test of Cure (TOC) in the microbiologic Intent-to-Treat (microITT) population. We determined outcomes by pathogen resistance phenotype and genotype among patients in the CERTAIN-1 study, with a focus on the extended microITT population that included all patients in the microITT population plus patients with pathogens that were resistant to either cefepime-taniborbactam or meropenem.

## MATERIALS AND METHODS

### Trial design and oversight

CERTAIN-1 was a randomized, double-blind, Phase 3 cUTI study. The primary objective was to evaluate the efficacy of cefepime-taniborbactam compared with meropenem in adult patients with cUTI, including acute pyelonephritis (AP). Details of trial design, oversight, eligibility criteria, and blinding have been previously reported (13).

### Randomization and treatment

Patients were randomly assigned 2:1 to receive cefepime-taniborbactam 2 g/0.5 g intravenously every 8 h over 2 h or meropenem 1 g intravenously every 8 h over 30 min for 7 days (up to 14 days for patients with concurrent bacteremia). Oral step-down therapy was not permitted.

### Identification and susceptibility testing of pathogens

Urine specimens were obtained at Screening, End of Therapy (EOT; within 24 h after the last dose of intravenous therapy), TOC (Day 19**–**23), and Late Follow-up (LFU; Day 28**–**35) visits for culture. Blood specimens were also collected at Screening and to document clearance.

Susceptibility testing of confirmed pathogens was performed centrally with concurrent quality control (13). Resistant phenotypes were based on Clinical and Laboratory Standards Institute (CLSI) breakpoints (14). Cefepime-taniborbactam MICs were interpreted with a provisional susceptible breakpoint of ≤16 µg/mL (12). An MDR phenotype corresponded to resistance to ≥1 agent in ≥3 classes of antibacterial agents.

### Prespecified analysis population, end points, and efficacy assessments

The extended microITT population included all patients with a qualifying gram-negative uropathogen(s) at ≥10^5^ CFU/mL, against which one or both study drugs had antibacterial activity [where activity was defined as a cefepime-taniborbactam MIC ≤16 µg/mL and a meropenem MIC ≤2 µg/mL (for Enterobacterales) or ≤4 µg/mL (for *P. aeruginosa*)], and no more than two microorganisms were identified regardless of colony count (13).

Microbiologic success required eradication of all baseline gram-negative uropathogen(s) to <10^3^ CFU/mL at TOC. Per-patient microbiologic response did not consider gram-positive pathogens. Gram-negative pathogens that were isolated at TOC were considered persistent for the present, prespecified analyses only if they matched the baseline pathogen clonal type, because isolation of a genotypically different pathogen at TOC indicated an infection by a distinct isolate compared with the baseline pathogen. Accordingly, patients with a uropathogen at ≥10^3^ CFU/mL at TOC that was clonally unrelated to the baseline pathogen were assessed with a microbiologic response of eradication. Clonal relatedness was assessed by multilocus sequence typing (MLST) or, for *Proteus mirabilis*, pulsed field electrophoresis (PFGE).

Clinical success was defined as patient was alive, had symptomatic resolution or return to premorbid baseline of all core cUTI signs and symptoms, had no new core cUTI signs or symptoms, and had no use of additional antibacterial agents for cUTI. Composite success required both microbiologic and clinical success.

### Genotypic analyses for β-lactam resistance mechanisms

Isolates of Enterobacterales with aztreonam, cefepime, ceftazidime, imipenem, and/or meropenem MIC ≥2 µg/mL, and isolates of *P. aeruginosa* with ceftazidime MICs of ≥16 µg/mL and/or imipenem/meropenem MIC of ≥2 µg/mL were subjected to whole genome sequencing (WGS) (see the supplemental material).

Assembled sequences were analyzed by proprietary software to align them against β-lactamase–encoding genes present in a curated database (15). AmpC gene variant and expression levels were determined for chromosomal *ampC* in *Serratia* spp. and *Enterobacter* spp., chromosomal *bla*_PDC_ in *P. aeruginosa*, and plasmidic *bla*_CMY_ in *Klebsiella pneumoniae*. Major porin gene sequences (among *E. coli*, *Klebsiella* spp., *Enterobacter* spp. and *P. aeruginosa*), efflux regulatory gene sequences (among *P. aeruginosa*), and the sequence of the *ftsI* gene encoding penicillin-binding protein 3 (PBP3; among *E. coli* and *P. aeruginosa*) were compared with reference alleles for qualifying isolates.

Post-baseline isolates with increases in study drug MIC of ≥4 fold compared with baseline were subjected to WGS analysis to assess potential resistance mechanisms.

## RESULTS

### Study population

The prespecified analyses reported here were performed in the extended microITT population (*n* = 452) that included all patients in the microITT population (*n* = 436) plus 16 patients with a baseline gram-negative uropathogen, against which only one study drug had an *in vitro* activity. The extended microITT population, therefore, included pathogens with diverse resistance profiles that were expected to be effectively treated by cefepime-taniborbactam and/or meropenem.

### Baseline pathogen species

All patients in the extended microITT population had at least one gram-negative uropathogen at baseline. The most common uropathogen overall was *E. coli* (66.8% of patients) followed by *K. pneumoniae* (15.3%), *P. aeruginosa* (5.1%), *P. mirabilis* (4.4%), and *Enterobacter cloacae* complex (3.8%) (Table S1).

### Resistance profiles in baseline Enterobacterales

Among baseline Enterobacterales pathogens (*n* = 437), 24.3%, 38.2%, and 2.3% were cefepime-, MDR-, and carbapenem-resistant, respectively (Table 1). Cefepime-taniborbactam at ≤16 µg/mL inhibited 99.8% of Enterobacterales overall (Table 1; Table S2). Taniborbactam decreased the cefepime MIC_90_ by 2,048-fold (to 0.25 µg/mL) against Enterobacterales overall, by ≥1,024 fold (to 1 µg/mL) against cefepime resistant and MDR subsets of Enterobacterales, and by ≥128 fold (to 8 µg/mL) against CRE.

**TABLE 1:** Susceptibility summary for baseline isolates of Enterobacterales and *P. aeruginosa* (extended MicroITT population)^*f*^

	MIC_90_ or MIC range (µg/mL); % Susceptible						
Pathogen phenotype/genotype (n; % of total)^*a*^	Cefepime	Cefepime-taniborbactam^*b*^	Meropenem	Ceftazidime-avibactam	Ceftolozane-tazobactam	Meropenem-vaborbactam^*e*^	Piperacillin- tazobactam
All Enterobacterales (437; 100%)	512; 73.5	0.25; 99.8	0.12; 96.8	0.5; 99.3	4; 89.7	0.06; 98.2	64; 86.0
Cefepime resistant (106; 24.3%)	>512; 0	1; 99.1	2; 88.7	1; 97.2	>64; 60.4	0.5; 93.4	>128; 60.4
MDR^*c*^ (167; 38.2%)	>512; 34.7	1; 99.4	0.5; 91.6	1; 98.2	>64; 73.1	0.12; 95.2	>128; 67.1
Carbapenem resistant (10; 2.3%)	>512; 10.0	8; 100	64; 0	>64; 70.0	>64; 0	64; 30.0	>128; 0
ESBL genotype^*g*^ (117; 26.8%)	>512; 5.1	1; 99.1	1; 91.5	1; 97.4	>64; 65.0	0.25; 94.0	>128; 64.1
AmpC^*d*^ (18; 4.1%)	>512; 22.2	4; 100	32; 83.3	>64; 83.3	>64; 38.9	32; 83.3	>128; 38.9
Carbapenemase (13; 3.0%)	>512; 7.7	16; 92.3	64; 23.1	>64; 76.9	>64; 0	64; 46.2	>128; 0
All *P. aeruginosa* (23; 100%)	32; 69.6	16; 91.3	16; 82.6	32; 82.6	>64; 87.0	16; 87.0	>128; 60.9
Cefepime resistant (6; 26.1%)	32–512; 0	4–32; 66.7	0.25 to >64; 50.0	8–64; 33.3	2 to >64; 50.0	0.25 to >64; 50.0	32 to >128; 0
MDR^*c*^ (7; 30.4%)	16–512; 0	4–32; 71.4	0.25 to >64; 57.1	4–64; 42.9	1 to >64; 57.1	0.25 to >64; 57.1	32 to >128; 0
Carbapenem resistant (5; 21.7%)	4–512; 40.0	4–16; 100	0.5 to >64; 20.0	1–64; 60.0	0.5 to >64; 60.0	0.25 to >64; 40.0	1–128; 40.0
Carbapenemase (1; 4.3%)	32; 0	8; 100	>64; 0	64; 0	>64; 0	>64; 0	64; 0

^*a*^ Patients had at least one gram-negative uropathogen at baseline and not more than two uropathogens in total.

^*b*^ In the absence of breakpoints for cefepime-taniborbactam, % Susceptible corresponds to the percentage of isolates inhibited at ≤16 µg/mL (12).

^*c*^ Resistance to ≥1 agent in ≥3 classes of antibacterial agents.

^*d*^ Includes pathogens carrying plasmidic *ampC* and pathogens at high risk of clinically significant AmpC production due to an inducible *ampC* gene (*E. cloacae* complex, *Klebsiella aerogenes*, and *Citrobacter freundii*; 7).

^*e*^ Meropenem-vaborbactam MICs against *P. aeruginosa* were interpreted using the EUCAST susceptible breakpoint of ≤8 µg/mL.

^*f*^ Underlined values correspond to the percentage of isolates in each phenotypic or genotypic subset that are susceptible to the given agent.

^*g*^ Abbreviation: ESBL, extended spectrum β-lactamase genotype.

Overall, 26.8%, 4.1%, and 3.0% of baseline Enterobacterales isolates carried genes for ESBLs, AmpC, and/or carbapenemases, respectively (Table 1). Taniborbactam decreased the cefepime MIC_90_ by ≥1,024 fold (to 1 µg/mL) against Enterobacterales carrying ESBL genes, by ≥256 fold (to 4 µg/mL) against the AmpC subset of isolates, and by ≥64 fold (to 16 µg/mL) against isolates carrying carbapenemase genes. Cefepime-taniborbactam at ≤16 µg/mL inhibited 92.3% of Enterobacterales isolates carrying carbapenemase genes and 99.1%–100% of Enterobacterales isolates in the other defined resistant genotypic subsets. These percentages were higher than the corresponding rates of susceptibility to all tested comparators.

Among 123 characterized isolates of Enterobacterales, 117 (95.1%) carried one or more ESBL genes, primarily *bla*_CTX-M_ (in 111 isolates; 79 of which carried *bla*_CTX-M-15_) and *bla*_OXA-1/OXA-1-like_ (in 53 isolates) (Table 2).

**TABLE 2:** Summary of β-lactamase gene content and cefepime, cefepime-taniborbactam, and meropenem MICs for characterized baseline isolates of Enterobacterales and *P. aeruginosa* (extended microITT population)

Organism	Carbapenemase	ESBL	AmpC^*a*^	*N*	MIC or MIC range (µg/mL)		
					Cefepime	Cefepime-taniborbactam	Meropenem
*C. freundii* complex				1	4	0.06	≤0.03
	–^*j*^	–	CMY-70	1	4	0.06	≤0.03
*E. cloacae* complex				9	1 to >512	0.12–4	≤0.03–0.12
	–	–	ACT-30-like	1	1	1	≤0.03
	–	CTX-M-15	ACT-17	1	>512	0.12	0.06
	–	CTX-M-15; CTX-M-3; OXA-1	ACT-24	2	>512	0.5–1	0.06–0.12
	–	CTX-M-15; OXA-1	ACT-17	1	>512	1	0.12
		CTX-M-15; OXA-1	ACT-24	2	>512	0.5–2	0.12
		CTX-M-15; OXA-1	ACT-30-like	1	>512	4	0.06
	–	CTX-M-15; OXA-1-like	ACT-45	1	>512	0.12	≤0.03
*E. coli*				68	0.03 to >512	≤0.008–32	≤0.03–0.12
	OXA-181	CTX-M-55; OXA-1-like	–	1^*b*^	>512	32	1
	–	CTX-M-1	–	2	8–512	0.03–0.06	≤0.03
	–	CTX-M-1; CTX-M-55	–	1	>512	0.06	≤0.03
	–	CTX-M-14	–	1	2	0.06	≤0.03
	–	CTX-M-14; CTX-M-14	–	1	512	0.06	≤0.03
	–	CTX-M-15	–	13	1 to 512	≤0.008–0.25	≤0.03–0.06
	–	CTX-M-15	DHA-1	1	512	0.06	≤0.03
	–	CTX-M-15; OXA-1	–	22	0.06 to >512	0.016–0.5	≤0.03–0.06
	–	CTX-M-15; OXA-1-like	–	1	512	0.12	≤0.03
	–	CTX-M-27	–	8	8–128	0.03–0.12	≤0.03
	–	CTX-M-3	–	5	16–512	0.06–0.12	≤0.03
	–	CTX-M-55	–	5	32 to >512	0.03–0.5	≤0.03–0.12
	–	CTX-M-64	–	1	16	0.06	≤0.03
	–	CTX-M-65	–	3	4–32	0.03–0.12	≤0.03
	–	CTX-M-71; OXA-1	–	1	128	0.5	≤0.03
	–	–	CMY-2	1	0.25	0.06	≤0.03
	–	–	DHA-1	1	≤0.03	0.06	≤0.03
*K. aerogenes*				1	0.06	0.12	0.06
	–	–	AmpC-like	1	0.06	0.12	0.06
*Klebsiella oxytoca*				1	32	0.12	0.06
	–	CTX-M-3; OXY-1–2	–	1	32	0.12	0.06
*K. pneumoniae*				39	1 to >512	0.03–4	≤0.03 to >64
	KPC-3	–	–	2	256	0.5–1	64 to >64
	NDM-1	CTX-M-15	CMY-4↑	2	512 to >512	1–4	16–32
	OXA-232	CTX-M-15; OXA-1	–	1	>512	8	64
	OXA-48	–	–	1	1	0.12	4
	OXA-48	CTX-M-15	–	2	>512	0.5	4–8
	OXA-48	CTX-M-15; OXA-1	–	3	32 to >512	0.5–2	1–32
	–	CTX-M-15	–	10	16–512	0.06–4	≤0.03–2
	–	CTX-M-15; OXA-1	–	14	16 to >512	0.03–1	≤0.03–0.25
	–	CTX-M-15; SHV-27	–	1	8	0.03	≤0.03
	–	OXA-1	–	1	2	0.5	≤0.03
	–	OXA-1	CMY-4↑	1	8	4	0.12
	–	SHV-12	–	1	64	1	0.25
*P. mirabilis*				5	4 to >512	0.03–0.5	0.06–0.12
	–	CTX-M-15; OXA-1; VEB-6	–	1	256	0.5	0.06
	–	CTX-M-55	–	1	>512	0.06	0.12
	–	TEM-92	–	1	4	0.03	0.12
	–	VEB-16	–	1	64	0.25	0.06
	–	VEB-6	–	1	32	0.06	0.06
*P. aeruginosa^c^*				14	1–512	1–32	0.25 to >64
	VIM-2	–	PDC-3	1^*d*^	32	8	>64
	–	GES-19	PDC-19a	1*^d^**^,^^e^^,^^h^*	256	4	0.25
	–	VEB-9	PDC-11	1*^d^**^,^^f^*	512	8	16
	–	–	PDC-1	1*^d^**^,^^e^*	1	1	0.5
	–	–	PDC-1↑	1*^d^*^,^*^e^*^,^^*h*^	32	16	16
	–	–	PDC-1↑	1*^d^**^,^^e^*	32	32	2
	–	–	PDC-3	1	2	2	0.25
	–	–	PDC-3	1^*h*^	2	2	0.25
	–	–	PDC-3	1^*d*^	4	4	0.25
	–	–	PDC-5↑	1^*d*^^,^^*e*^^,*g*^	4	4	0.5^*i*^
	–	–	PDC-8	1^*f*^	8	8	2
	–	–	PDC-28↑	1^*d*,*f*,*h*^	32	32	1
	–	–	PDC-31	1^*d*^	8	4	2
	–	–	PDC-98	1^*d*^^,*e*^	16	16	1
*Serratia marcescens*				1	256	16	64
	NDM-1	CTX-M-3	DHA-1-like	1	256	16	64

^*a*^ ↑ symbol indicates *ampC* gene was overexpressed relative to reference.

^*b*^ Isolate possessed a YRIN insertion in penicillin binding protein 3 associated with cephalosporin and monobactam resistance (16).

^*c*^ All isolates possessed genes for intrinsic oxacillinase and PA5542 /PA5542-like β-lactamases.

^*d*^ Isolate possessed disruptions or alterations to *oprD*.

^*e*^ Isolate possessed overexpressed MexXY-OprM.

^*f*^ Isolate possessed overexpressed MexAB-OprM.

^*g*^ Isolate possessed overexpressed MexCD-OprJ.

^*h*^ Isolate possessed alterations to *ftsI*/PBP3 gene.

^*i*^ Imipenem MIC, 16 µg/mL**.**

^*j*^ –, not identified.

Plasmidic AmpC genes included CMY-4 (in three isolates of *K. pneumoniae*; all of which overexpressed the gene), CMY-2 (in one isolate of *E. coli*), and DHA-1/DHA-1-like (in two isolates of *E. coli*, and one isolate of *S. marcescens*). Intrinsic AmpC genes (AmpC type [ACT] variants in nine isolates of *E. cloacae* complex; CMY-70 in one isolate of *C. freundii*; and AmpC-like in one isolate of *K. aerogenes*; Table 2) were expressed at basal levels among characterized isolates.

Overall, 13 baseline Enterobacterales isolates carried genes for OXA-48 group (in 7 isolates of *K. pneumoniae* and 1 isolate of *E. coli*), NDM-1 (in 2 isolates of *K. pneumoniae* and 1 isolate of *S. marcescens*), and KPC-3 (in 2 isolates of *K. pneumoniae*) carbapenemases (Table 2).

One isolate of *E. coli* (cefepime MIC, >512 µg/mL; cefepime-taniborbactam MIC, 32 µg/mL) carried genes for CTX-M-55 and OXA-1-like ESBLs and OXA-181 carbapenemase (Table 2), and had a 4-amino acid (YRIN) insertion in PBP3 associated with reduced susceptibility to cephalosporins and monobactams (16).

Among 118 baseline isolates of *E. coli*, *Klebsiella* spp., and *E. cloacae* complex with characterized porin sequences, 116 (98.3%) had altered or disrupted genes encoding OmpC/OmpK36, and 112 (94.9%) had altered or disrupted genes encoding OmpF/OmpK35. Except for the *E. coli* isolate carrying an insertion within PBP3 described above, cefepime-taniborbactam MICs were ≤0.5 µg/mL against *E. coli* isolates with porin alterations or disruptions. Similarly, cefepime-taniborbactam MICs were ≤2 µg/mL against 35/39 (89.7%) isolates of *K. pneumoniae* with porin alterations or disruptions. The remaining four characterized *K. pneumoniae* isolates had cefepime-taniborbactam MICs of 4–8 µg/mL (cefepime MIC range, 8–>512 µg/mL; meropenem MIC range, 0.12–64 µg/mL) and genes for CTX-M-15 and/or OXA-1 ESBLs; two also had overexpressed CMY-4; and two also carried carbapenemase genes (NDM-1 or OXA-232) (Table 2). Among the nine characterized isolates of *E. cloacae* complex, five had alterations, and four had disruptions in OmpF, and all nine had alterations in OmpC. Corresponding cefepime-taniborbactam and meropenem MIC ranges were 0.12–4 µg/mL and ≤0.03–0.12 µg/mL, respectively.

### Resistance profiles in baseline *P. aeruginosa*

Among baseline *P. aeruginosa*, 26.1%, 30.4%, and 21.7% of isolates were cefepime-, MDR-, and carbapenem-resistant, respectively (Table 1). Cefepime-taniborbactam at ≤16 µg/mL inhibited 91.3% of *P. aeruginosa* overall and 66.7%, 71.4% and 100% of isolates in these resistant subsets of *P. aeruginosa*, respectively. These percentages were higher than the corresponding rates of susceptibility to all tested comparators.

Acquired ESBL genes were identified in 2 of the 14 characterized *P. aeruginosa* isolates (Table 2). One isolate carried *bla*_GES-19_, showed moderate overexpression of *mexX*, had OprD alterations, had a predicted N117S amino acid substitution within PBP3, and had cefepime, cefepime-taniborbactam, and meropenem MICs of 256, 4, and 0.25 µg/mL, respectively. The other isolate carried *bla*_VEB-9_, showed moderate overexpression of *mexA*, had disrupted OprD, and had cefepime, cefepime-taniborbactam, and meropenem MICs of 512, 8, and 16 µg/mL, respectively.

One *P. aeruginosa* isolate carried a gene for VIM-2 metallo-β-lactamase, had disrupted OprD, and had cefepime, cefepime-taniborbactam, and meropenem MICs of 32, 8, and >64 µg/mL, respectively (Table 2).

Overexpression of *bla*_PDC_ and efflux pump genes (*mexA*, *mexC*, and/or *mexX*) was observed in 4 and 7 strains, respectively, among the other 11 characterized *P. aeruginosa* isolates (Table 2). Seven strains (63.6%) were susceptible to cefepime, 9 (81.8%) were inhibited by ≤16 µg/mL cefepime-taniborbactam, and 10 (90.9%) were susceptible to meropenem. Three of these 11 isolates of *P. aeruginosa* also had predicted alterations in PBP3. The *ftsI* gene in one isolate with cefepime-taniborbactam MIC of 2 µg/mL encoded PBP3 with a N117S substitution. A second isolate, with cefepime-taniborbactam and meropenem MICs of 16 µg/mL, had a G531D substitution within PBP3; a premature truncation (K56X) within OprD; and moderately (8.3-fold) and highly (371.3-fold) overexpressed MexX and PDC-1, respectively. The third isolate, with cefepime and cefepime-taniborbactam MICs of 32 µg/mL, overexpressed PDC-28 by 44-fold, had MexAB-OprM overexpressed by 10.9-fold, carried numerous predicted amino acid alterations within OprD, and had a predicted A139S substitution within PBP3.

### Resistance profiles in baseline Enterobacterales and *P. aeruginosa* pathogens exclusive to the extended microITT population

In the subset of 16 patients in the extended microITT population that were excluded from the microITT population owing to resistance to one study drug, 3 patients (2 in the cefepime-taniborbactam group and 1 in the meropenem group) had baseline pathogens with cefepime-taniborbactam MICs of 32 µg/mL (meropenem MIC range, 1–2 µg/mL) and the remaining 13 patients (10 in the cefepime-taniborbactam group, 3 in the meropenem group) had pathogens that were resistant to meropenem (meropenem MIC range, 4–>64 µg/mL; cefepime-taniborbactam MIC range, 0.12–16 µg/mL) (Table 3). All 16 baseline pathogens were MDR and 15/16 were resistant to cefepime. Among the cefepime-resistant pathogens, taniborbactam decreased the cefepime MIC by 16-fold against *S. marcescens*, by ≥32 fold against *E. coli*, by up to ≥2,048 fold against *K. pneumoniae*, and by up to ≥64 fold against *P. aeruginosa*. Overall, taniborbactam restored cefepime MICs to ≤16 µg/mL (provisionally Susceptible) for 13/15 of the cefepime-resistant isolates.

**TABLE 3:** Phenotypic and genotypic profiles of baseline pathogens, and composite, microbiologic, and clinical outcomes at TOC, for patients in the extended microITT population and excluded from the microITT population, by treatment group^*f*^^,*g*^

		Minimum inhibitory concentration (µg/mL) (Interpretation^*a*^)								Response at TOC (Day 19–23)		
**Patient**	**Baseline pathogen**	**FEP**	**FTB^*b*^**	**MEM**	**CZA**	**CT**	**MEV^*c*^**	**TZP**	**β-lactam resistance mechanisms^*d*^^,^^*e*^**	**Composite**	**Microbiologic**	**Clinical**
Cefepime-taniborbactam treatment group												
A	*K. pneumoniae*	1 (S)	0.12 (S)	4 (R)	0.25 (S)	4 (I)	4 (S)	>128 (R)	**OXA-48**; OmpK36 disrupted; OmpK35 disrupted	Responder	Success	Success
B	*K. pneumoniae*	256 (R)	0.5 (S)	>64 (R)	1 (S)	>64 (R)	0.25 (S)	>128 (R)	**KPC-3**; OmpK36 disrupted; OmpK35 disrupted	Responder	Success	Success
C	*K. pneumoniae*	>512 (R)	0.5 (S)	4 (R)	2 (S)	>64 (R)	8 (I)	>128 (R)	**OXA-48**; CTX-M-15; OmpK36 disrupted; OmpK35 alterations	Responder	Success	Success
D	*K. pneumoniae*	>512 (R)	0.5 (S)	8 (R)	0.5 (S)	>64 (R)	8 (I)	>128 (R)	**OXA-48**; CTX-M-15; OmpK36 disrupted; OmpK35 alterations	Responder	Success	Success
E	*K. pneumoniae*	256 (R)	1 (S)	64 (R)	2 (S)	>64 (R)	0.25 (S)	>128 (R)	**KPC-3**; OmpK36 disrupted; OmpK35 disrupted	Responder	Success	Success
F	*K. pneumoniae*	>512 (R)	4 (S)	32 (R)	>64 (R)	>64 (R)	32 (R)	>128 (R)	**NDM-1**; CTX-M-15; CMY-4↑; OmpK36 disrupted; OmpK35 disrupted	Responder	Success	Success
G	*P. aeruginosa*	32 (R)	8 (S)	>64 (R)	64 (R)	>64 (R)	>64 (R)	64 (I)	**VIM-2**; PDC-3; OXA-486; PA5542-like; OprD disrupted	Responder	Success	Success
H	*P. aeruginosa*	512 (R)	8 (S)	16 (R)	64 (R)	>64 (R)	16 (R)	32 (I)	PDC-11; VEB-9; OXA-846; PA5542-like; MexAB-OprM↑; OprD disrupted	Non-responder	Failure	Failure
I	*K. pneumoniae*	>512 (R)	8 (S)	64 (R)	2 (S)	>64 (R)	64 (R)	>128 (R)	**OXA-232**; CTX-M-15; OXA-1; OmpK36 disrupted; OmpK35 alterations	Non-responder	Failure	Success
J	*S. marcescens*	256 (R)	16 (S)	64 (R)	>64 (R)	>64 (R)	64 (R)	>128 (R)	**NDM-1**; CTX-M-3; DHA-1-like; SRT-2;	Responder	Success	Success
K	*P. aeruginosa*	32 (R)	32 (R)	2 (S)	32 (R)	2 (S)	2 (S)	128 (R)	PDC-1↑; OXA-847; PA5542-like; MexXY-OprM↑; OprD alterations	Responder	Success	Success
L	*P. aeruginosa*	32 (R)	32 (R)	1 (S)	8 (S)	4 (S)	2 (S)	>128 (R)	PDC-28↑; OXA-846-like; PA5542-like; MexAB-OprM↑; OprD alterations; PBP3 alterations	Non-responder	Failure	Success
Meropenem treatment group												
M	*E. coli*	>512 (R)	32 (R)	1 (S)	2 (S)	>64 (R)	1 (S)	>128 (R)	**OXA-181**; CTX-M-55; OXA-1; OmpF alterations; OmpC disrupted; PBP3 insertion YRIN	Non-responder	Failure	Failure
N	*P. aeruginosa*	32 (R)	16 (S)	16 (R)	8 (S)	4 (S)	16 (R)	128 (R)	PDC-1↑, PA5542-like; OprD disrupted; MexXY-OprM↑; PBP3 alteration G531D	Responder	Success	Success
O	*K. pneumoniae*	512 (R)	1 (S)	16 (R)	>64 (R)	>64 (R)	16 (R)	>128 (R)	**NDM-1**; CTX-M-15; CMY-4↑; OmpK36 disrupted; OmpK35 disrupted	Responder	Success	Success
P	*K. pneumoniae*	256 (R)	2 (S)	32 (R)	1 (S)	>64 (R)	32 (R)	>128 (R)	**OXA-48**; CTX-M-15; OXA-1; OmpK36 disrupted; OmpK35 disrupted	Responder	Success	Success

^*a*^ MICs were interpreted using CLSI breakpoints (14).

^*b*^ MICs were interpreted using proposed breakpoints of Susceptible ≤16 µg/mL, Resistant ≥32 µg/mL based on 12.

^*c*^ MICs against *P. aeruginosa* were interpreted using the EUCAST susceptible breakpoint of ≤8 µg/mL.

^*d*^ ↑Indicates overexpression of intrinsic or acquired *ampC* gene or efflux regulatory gene relative to reference wild-type strain.

^*e*^ Carbapenemases are indicated in bold font; metallo-β-lactamases are underlined.

^*f*^ Rows are ordered from lowest to highest MIC to study drug received in the corresponding treatment group.

^*g*^ Abbreviations: FEP, cefepime; FTB, cefepime-taniborbactam; MEM, meropenem; CZA, ceftazidime-avibactam; CT, ceftolozane-tazobactam; MEV, meropenem-vaborbactam; TZP, piperacillin-tazobactam; S, susceptible; I, intermediate; R, resistant.

### Composite, microbiologic, and clinical outcomes by resistance phenotype and genotype

In the subset of 16 patients who were exclusive to the extended microITT population, cefepime-taniborbactam demonstrated composite, microbiological, and/or clinical successes in patients with baseline pathogens having cefepime-taniborbactam MICs up to and including 32 µg/mL and meropenem MICs up to and including >64 µg/mL (Table 3). Eight of the 10 cefepime-taniborbactam-treated patients (80.0%) with meropenem-resistant baseline pathogens achieved composite success whereas 9 (90.0%) achieved clinical success. Meropenem-treated patients achieved composite success in the three instances with meropenem-resistant pathogens. Furthermore, cefepime-taniborbactam-treated patients achieved composite success in 5/6 cases (83.3%) and clinical success in all six instances of patients with pathogens carrying serine carbapenemase genes (two isolates of *K. pneumoniae* with KPC-3, three isolates of *K. pneumoniae* with OXA-48, one isolate of *K. pneumoniae* with OXA-232) and in 3/3 patients (100%) with pathogens carrying metallo-β-lactamase genes (one *K*. *pneumoniae*, one *S*. *marcescens* each with NDM-1; one isolate of *P. aeruginosa* with VIM-2).

Three patients (two in the cefepime-taniborbactam group, one in the meropenem group) had meropenem-susceptible baseline pathogens with cefepime-taniborbactam MICs of 32 µg/mL (Table 3). One of these cefepime-taniborbactam patients achieved composite success and the other was a composite non-responder whereas both achieved clinical success. The meropenem patient was not only a composite non-responder but also a microbiologic and clinical failure.

Within each treatment group of the extended microITT population overall, composite success rates at TOC in resistant subsets by pathogen species were generally similar to composite success rates by corresponding pathogen overall (Table S3). Two exceptions were in the meropenem group, where composite success rates were numerically lower for patients with cefepime-resistant *E. coli* (9/19; 47.4%) and ESBL *E. coli* (13/25; 52.0%) compared with *E. coli* overall (62/100; 62.0%).

Where there were 10 or more patients within each treatment group to permit between-group comparisons by resistance subset, composite and microbiologic success rates at TOC in the extended microITT population were numerically higher for cefepime-taniborbactam compared with meropenem for Enterobacterales overall, for *E. coli* overall, for cefepime-resistant, MDR, and ESBL *E. coli*, and for *P. mirabilis* overall (Table S3). Meropenem patients had numerically higher composite success rates in cefepime-resistant, MDR, and ESBL subsets of *K. pneumoniae*. Clinical success rates across pathogens and resistance subsets were generally similar within and between treatment groups.

Cefepime-taniborbactam achieved composite success at TOC in 8/16 patients (50.0%) and clinical success in 13/16 patients (81.3%) with *P. aeruginosa*, respectively; corresponding rates were 4/7 (57.1%) and 6/7 (85.7%) for meropenem (Table 3). Within each treatment group, success rates at TOC in patients with resistant isolates of *P. aeruginosa* were similar to those against *P. aeruginosa* overall; however, modest numbers of patients limited further interpretation. The one patient in the cefepime-taniborbactam group with *P. aeruginosa* carrying a metallo-β-lactamase (VIM-2) gene (Table 2; Table 3) achieved composite success at TOC. Other microbiologic outcomes are described in the supplemental material.

## DISCUSSION

In the CERTAIN-1 study, substantial percentages of patients had baseline Enterobacterales pathogens in key resistance categories, consistent with the high burden of antimicrobial resistance globally (3, 5, 12, 17–19). Carriage of ESBL genes and an MDR phenotype were particularly prevalent in isolates of *E. cloacae* complex (47.1% and 64.7%, respectively) and *K. pneumoniae* (52.2% and 63.8%, respectively), reflecting the propensity of these organisms to acquire diverse resistance mechanisms. These observations, together with the finding that the most common uropathogen, *E. coli*, frequently carried ESBL genes or had an MDR phenotype underscore the need for active empiric and directed antibacterial agents for patients with cUTI.

Taniborbactam substantially potentiated cefepime *in vitro* activity against most isolates of Enterobacterales and *P. aeruginosa* that were cefepime-, multidrug-, or carbapenem-resistant, or that carried genes for ESBLs, AmpC enzymes or carbapenemases. These results are consistent with studies showing that taniborbactam restores cefepime activity against cefepime-, multidrug-, and carbapenem-resistant gram-negative pathogens producing serine-, NDM-, and VIM metallo-β-lactamases (9–12).

Within each treatment group, rates of composite success at TOC in each resistance subset were generally similar to rates of composite success overall. However, meropenem composite success rates in patients with Enterobacterales overall and within the most prevalent resistance subsets of Enterobacterales were numerically lower than corresponding cefepime-taniborbactam composite success rates. Whether patient, microbiologic, or pharmacokinetic factors could explain numeric differences in rates of composite success at TOC between treatment groups for these patients, including the subset of patients with ESBL *E. coli* at baseline [cefepime-taniborbactam, 31/41 (75.6%); meropenem, 13/25 (52.0%)] remains to be determined. For example, it is unknown whether these lower composite response rates among meropenem patients could be attributed to differences in urinary pharmacokinetic parameters between the study drugs, as has been posited recently for tebipenem pivoxil compared with ertapenem (20, 21).

Outcomes in patients with *P. aeruginosa* warrant particular attention as this gram-negative pathogen is frequently associated with nosocomial infections, treatment failures and antimicrobial resistance worldwide. In a recent global surveillance study, ceftazidime-avibactam and ceftolozane-tazobactam had *in vitro* activity against only 75% of carbapenemase-negative strains of CRPA, less than 50% of MDR *P. aeruginosa* strains, and <15% of carbapenemase-producing strains of CRPA (12). Hence, there is an unmet need for effective agents against resistant subsets of this challenging organism. Cefepime-taniborbactam at ≤16 µg/mL inhibited 4/4, 5/7, and 1/1 of these resistant *P. aeruginosa* strains, respectively, from patients in CERTAIN-1. These results, while limited, are consistent with corresponding percentages of 94.6%, 81.4%, and 62.2% against these resistant subsets, respectively, in global surveillance (12).

As also seen in other randomized controlled trials in adults with cUTI (20, 22–25), clinical success rates in the CERTAIN-1 study were higher than microbiologic and composite (i.e., microbiologic plus clinical) success rates for both treatment groups. Whereas asymptomatic bacteriuria was associated with a higher rate of late clinical failure in a recent analysis of cUTI trial data (26), bacteriuria in the absence of clinical signs and symptoms has only modest sensitivity and poor positive predictive value as a biomarker for late clinical failure (27). Withholding antibiotic treatment for most patients with asymptomatic bacteriuria is consistent with current guidelines (28).

Efficacy analyses by pathogen and by resistance subset in the Phase 3 CERTAIN-1 study demonstrated that *E. cloacae* complex, *E. coli*, *K. pneumoniae*, *P. aeruginosa*, and *P. mirabilis* are appropriate target organisms for cefepime-taniborbactam treatment. Cefepime-taniborbactam may offer a potential new carbapenem-sparing treatment option for patients with cUTI due to cefepime-, multidrug-, and CRE and *P. aeruginosa* including pathogens with ESBL, AmpC, and carbapenemase genes that have become commonplace worldwide.

## SUPPLEMENTAL MATERIAL

Supplemental material aac.00236-24-s0001.docx Tables S1 to S4 and supplemental methods and results.

ASM does not own the copyrights to Supplemental Material that may be linked to, or accessed through, an article. The authors have granted ASM a non-exclusive, world-wide license to publish the Supplemental Material files. Please contact the corresponding author directly for reuse.
