# Supplementary material for: Patient outcomes by baseline pathogen resistance phenotype and genotype in CERTAIN-1, a Phase 3 study of cefepime-taniborbactam versus meropenem in adults with complicated urinary tract infection
Source: Antimicrob Agents Chemother. 2024 May 23;68(7):e00236-24. doi: 10.1128/aac.00236-24 (PMC11232400; doi:10.1128/aac.00236-24)
Supplement: Supplemental material — Tables S1 to S4 and supplemental methods and results. [file aac.00236-24-s0001.docx]

Supplemental Material to:

Patient Outcomes by Baseline Pathogen Resistance Phenotype and Genotype in CERTAIN-1, a Phase 3 Study of Cefepime-Taniborbactam versus Meropenem in Adults with Complicated Urinary Tract Infection

Greg Moeck^a^#, Leanne B. Gasink^b^, Rodrigo E. Mendes^c^, Leah N. Woosley^c^, MaryBeth Dorr^a^, Hongzi Chen^a^, Florian M. Wagenlehner^d^, Tim Henkel^a^, Paul C. McGovern^a^

^a^Venatorx Pharmaceuticals, Inc., Malvern, PA

^b^LBG Consulting, LLC, Saint Davids, PA

^c^JMI Laboratories, North Liberty, IA, USA

^d^Justus Liebig University, Giessen, Germany

Supplementary Table 1 Baseline pathogens among patients in the extended microITT population^1^

|  | Cefepime-taniborbactam N=305 | |  | Meropenem N=147 | |  | Total N=452 | |
| --- | --- | --- | --- | --- | --- | --- | --- | --- |
|  | n | % |  | n | % |  | n | % |
| All Pathogens | 305 | 100 |  | 147 | 100 |  | 452 | 100 |
| Gram negative pathogens | 305 | 100 |  | 147 | 100 |  | 452 | 100 |
| Enterobacterales | 289 | 94.8 |  | 40 | 95.2 |  | 429 | 94.9 |
| *Citrobacter freundii* complex | 2 | 0.7 |  | 2 | 1.4 |  | 4 | 0.9 |
| *Citrobacter koseri* | 1 | 0.3 |  | 1 | 0.7 |  | 2 | 0.4 |
| *Enterobacter cloacae* complex | 14 | 4.6 |  | 3 | 2.0 |  | 17 | 3.8 |
| *Escherichia coli* | 202 | 66.2 |  | 100 | 68.0 |  | 302 | 66.8 |
| *Klebsiella aerogenes* | 1 | 0.3 |  | 1 | 0.7 |  | 2 | 0.4 |
| *Klebsiella oxytoca* | 5 | 1.6 |  | 1 | 0.7 |  | 6 | 1.3 |
| *Klebsiella pneumoniae* | 47 | 15.4 |  | 22 | 15.0 |  | 69 | 15.3 |
| *Klebsiella variicola* | 1 | 0.3 |  | 1 | 0.7 |  | 2 | 0.4 |
| *Kluyvera ascorbata* | 1 | 0.3 |  | 0 | - |  | 1 | 0.2 |
| *Morganella morganii* | 2 | 0.7 |  | 2 | 1.4 |  | 4 | 0.9 |
| *Proteus hauseri* | 1 | 0.3 |  | 0 | - |  | 1 | 0.2 |
| *Proteus mirabilis* | 10 | 3.3 |  | 10 | 6.8 |  | 20 | 4.4 |
| *Proteus penneri* | 1 | 0.3 |  | 0 | - |  | 1 | 0.2 |
| *Proteus vulgaris* | 2 | 0.7 |  | 0 | - |  | 2 | 0.4 |
| *Providencia rettgeri* | 0 | - |  | 1 | 0.7 |  | 1 | 0.2 |
| *Serratia marcescens* | 3 | 1.0 |  | 0 | - |  | 3 | 0.7 |
| *Pseudomonas aeruginosa* | 16 | 5.2 |  | 7 | 4.8 |  | 23 | 5.1 |
| Gram positive pathogens | 2 | 0.7 |  | 1 | 0.7 |  | 3 | 0.7 |
| *Enterococcus faecalis* | 1 | 0.3 |  | 1 | 0.7 |  | 2 | 0.4 |
| *Staphylococcus saprophyticus* | 1 | 0.3 |  | 0 | - |  | 1 | 0.2 |

^1^The denominator for percentages is the number of patients in each treatment group. Patients may have more than one pathogen at baseline. Multiple isolates of the same pathogen (i.e. species) from the same patient are counted only once for each phenotype and once for the overall tabulation of pathogen.

Supplementary Table 2 In vitro susceptibility summary for baseline gram-negative pathogens (extended microITT population)

|  | MIC (µg/mL) | | | MIC_50_ (µg/mL) | MIC_90_ (µg/mL) | %S^1^ |
| --- | --- | --- | --- | --- | --- | --- |
| Organism/Agent (n) | Min | Max | Mode |  |  |  |
| Enterobacterales overall (437) | | | | | | |
| Ampicillin-sulbactam | 0.25/0.12 | >128/64 | 2/1 | 16/8 | 64/32 | 47.4 |
| Aztreonam | ≤0.03 | >64 | 0.12 | 0.12 | 64 | 73.5 |
| Cefepime | ≤0.03 | >512 | ≤0.03 | 0.06 | 512 | 73.5 |
| Cefepime-taniborbactam | ≤0.008 | 32 | 0.06 | 0.06 | 0.25 | 99.8 |
| Ceftazidime | ≤0.03 | >64 | 0.12 | 0.25 | 64 | 76.2 |
| Ceftazidime-avibactam | ≤0.03 | >64 | 0.12 | 0.12 | 0.5 | 99.3 |
| Ceftolozane-tazobactam | ≤0.03 | >64 | 0.25 | 0.25 | 4 | 89.7 |
| Imipenem | ≤0.03 | 64 | 0.12 | 0.12 | 1 | 93.1 |
| Levofloxacin | ≤0.008 | >16 | 0.06 | 0.25 | >16 | 58.1 |
| Meropenem | ≤0.03 | >64 | ≤0.03 | ≤0.03 | 0.12 | 96.8 |
| Meropenem-vaborbactam | ≤0.03/8 | 64/8 | ≤0.03/8 | ≤0.03/8 | 0.06/8 | 98.2 |
| Piperacillin-tazobactam | ≤0.12 | >128 | 2 | 2 | 64 | 86.0 |
| Tetracycline | 0.25 | >64 | >64 | 2 | >64 | 58.6 |
| Tobramycin | 0.06 | >64 | 1 | 1 | 32 | 76.4 |
| Trimethoprim-sulfamethoxazole | ≤0.03/0.6 | >64/1216 | 0.06/1.2 | 0.25/4.8 | >64/1216 | 63.8 |
| *Enterobacter cloacae* complex (17) | | | | | | |
| Ampicillin-sulbactam | 4/2 | >128/64 | 128/64 | 64/32 | >128/64 | 0 |
| Aztreonam | ≤0.03 | >64 | >64 | 1 | >64 | 52.9 |
| Cefepime | ≤0.03 | >512 | >512 | 2 | >512 | 52.9 |
| Cefepime-taniborbactam | 0.03 | 4 | 1 | 0.25 | 2 | 100 |
| Ceftazidime | 0.12 | >64 | >64 | 2 | >64 | 52.9 |
| Ceftazidime-avibactam | 0.06 | 1 | 1 | 1 | 1 | 100 |
| Ceftolozane-tazobactam | 0.25 | >64 | >64 | 2 | >64 | 58.8 |
| Imipenem | 0.12 | 1 | 0.5 | 0.5 | 0.5 | 100 |
| Levofloxacin | 0.03 | >16 | >16 | 16 | >16 | 35.3 |
| Meropenem | ≤0.03 | 0.12 | ≤0.03 | 0.06 | 0.12 | 100 |
| Meropenem-vaborbactam | ≤0.03/8 | 0.06/8 | ≤0.03/8 | ≤0.03/8 | 0.06/8 | 100 |
| Piperacillin-tazobactam | 1 | >128 | >128 | 16 | >128 | 52.9 |
| Tetracycline | 1 | >64 | >64 | 4 | >64 | 52.9 |
| Tobramycin | 0.5 | >64 | 0.5 | 16 | >64 | 47.1 |
| Trimethoprim-sulfamethoxazole | 0.06/1.2 | >64/1216 | 0.06/1.2 | 1/19 | >64/1216 | 58.8 |
| *Escherichia coli* (302) | | | | | | |
| Ampicillin-sulbactam | 0.25/0.12 | >128/64 | 2/1 | 8/4 | 64/32 | 51.3 |
| Aztreonam | ≤0.03 | >64 | 0.12 | 0.12 | 32 | 79.1 |
| Cefepime | ≤0.03 | >512 | ≤0.03 | 0.06 | 256 | 79.8 |
| Cefepime-taniborbactam | ≤0.008 | 32 | 0.06 | 0.06 | 0.12 | 99.7 |
| Ceftazidime | ≤0.03 | >64 | 0.12 | 0.25 | 16 | 82.5 |
| Ceftazidime-avibactam | ≤0.03 | 2 | 0.12 | 0.12 | 0.25 | 100 |
| Ceftolozane-tazobactam | ≤0.03 | >64 | 0.25 | 0.25 | 0.5 | 97.7 |
| Imipenem | ≤0.03 | 2 | 0.12 | 0.12 | 0.25 | 99.7 |
| Levofloxacin | ≤0.008 | >16 | 0.03 | 0.12 | 16 | 62.6 |
| Meropenem | ≤0.03 | 1 | ≤0.03 | ≤0.03 | ≤0.03 | 100 |
| Meropenem-vaborbactam | ≤0.03/8 | 1/8 | ≤0.03/8 | ≤0.03/8 | ≤0.03/8 | 100 |
| Piperacillin-tazobactam | ≤0.12 | >128 | 2 | 2 | 8 | 93.7 |
| Tetracycline | 0.25 | >64 | >64 | 2 | >64 | 60.6 |
| Tobramycin | 0.25 | >64 | 1 | 1 | 16 | 82.5 |
| Trimethoprim-sulfamethoxazole | ≤0.03/0.6 | >64/1216 | 0.06/1.2 | 0.12/2.4 | >64/1216 | 66.2 |
| *Klebsiella pneumoniae* (69) | | | | | | |
| Ampicillin-sulbactam | 4/2 | >128/64 | 8/4 | 32/16 | >128/64 | 31.9 |
| Aztreonam | ≤0.03 | >64 | >64 | 32 | >64 | 46.4 |
| Cefepime | ≤0.03 | >512 | >512 | 16 | >512 | 46.4 |
| Cefepime-taniborbactam | ≤0.008 | 8 | 0.06 | 0.12 | 2 | 100 |
| Ceftazidime | ≤0.03 | >64 | >64 | 16 | >64 | 46.4 |
| Ceftazidime-avibactam | ≤0.03 | >64 | 0.5 | 0.25 | 2 | 97.1 |
| Ceftolozane-tazobactam | 0.06 | >64 | 0.5 | 1 | >64 | 60.9 |
| Imipenem | 0.06 | 64 | 0.12 | 0.25 | 4 | 87.0 |
| Levofloxacin | 0.06 | >16 | >16 | 4 | >16 | 36.2 |
| Meropenem | ≤0.03 | >64 | ≤0.03 | ≤0.03 | 8 | 82.6 |
| Meropenem-vaborbactam | ≤0.03/8 | 64/8 | ≤0.03/8 | ≤0.03/8 | 4/8 | 91.3 |
| Piperacillin-tazobactam | 0.25 | >128 | >128 | 16 | >128 | 53.6 |
| Tetracycline | 1 | >64 | >64 | 2 | >64 | 65.2 |
| Tobramycin | 0.06 | >64 | 0.5 | 4 | >64 | 53.6 |
| Trimethoprim-sulfamethoxazole | 0.06/1.2 | >64/1216 | >64/1216 | 2/38 | >64/1216 | 50.7 |
| *Proteus mirabilis* (20) | | | | | | |
| Ampicillin-sulbactam | 0.5/0.25 | 32/16 | 1/0.5 | 1/0.5 | 32/16 | 80.0 |
| Aztreonam | ≤0.03 | >64 | ≤0.03 | ≤0.03 | 16 | 75.0 |
| Cefepime | ≤0.03 | >512 | 0.06 | 0.06 | 64 | 70.0 |
| Cefepime-taniborbactam | 0.03 | 0.5 | 0.06 | 0.06 | 0.25 | 100 |
| Ceftazidime | ≤0.03 | >64 | 0.06 | 0.06 | >64 | 80.0 |
| Ceftazidime-avibactam | ≤0.03 | 8 | ≤0.03 | ≤0.03 | 0.5 | 100 |
| Ceftolozane-tazobactam | 0.25 | 8 | 0.5 | 0.5 | 2 | 85.0 |
| Imipenem | 0.25 | 4 | 2 | 2 | 4 | 40.0 |
| Levofloxacin | 0.03 | >16 | 0.06 | 0.12 | >16 | 55.0 |
| Meropenem | 0.06 | 0.25 | 0.12 | 0.12 | 0.12 | 95.0 |
| Meropenem-vaborbactam | ≤0.03/8 | 0.12/8 | 0.12/8 | 0.12/8 | 0.12/8 | 95.0 |
| Piperacillin-tazobactam | ≤0.12 | 2 | 0.25 | 0.25 | 1 | 95.0 |
| Tetracycline | 32 | 64 | 32 | 32 | 64 | 0 |
| Tobramycin | 0.5 | >64 | 1 | 2 | 64 | 70.0 |
| Trimethoprim-sulfamethoxazole | ≤0.03/0.6 | >64/1216 | 0.06/1.2 | 0.12/2.4 | >64/1216 | 50.0 |
| *Pseudomonas aeruginosa* (23) | | | | | | |
| Aztreonam | 0.12 | >64 | 8 | 8 | 64 | 56.5 |
| Cefepime | 0.5 | 512 | 2 | 4 | 32 | 69.6 |
| Cefepime-taniborbactam | 0.5 | 32 | 2 | 4 | 16 | 91.3 |
| Ceftazidime | 0.25 | >64 | 2 | 4 | >64 | 69.6 |
| Ceftazidime-avibactam | 0.25 | 64 | 2 | 2 | 32 | 82.6 |
| Ceftolozane-tazobactam | 0.25 | >64 | 1 | 1 | >64 | 87.0 |
| Imipenem | 0.5 | >64 | 1 | 1 | 32 | 69.6 |
| Levofloxacin | 0.12 | >16 | >16 | 4 | >16 | 47.8 |
| Meropenem | ≤0.03 | >64 | 0.25 | 0.25 | 16 | 82.6 |
| Meropenem-vaborbactam | 0.06/8 | >64/8 | 0.25/8 | 0.25/8 | 16/8 | 87.0 |
| Piperacillin-tazobactam | 0.5 | >128 | 8 | 8 | >128 | 60.9 |
| Tobramycin | 0.25 | >64 | 1 | 1 | >64 | 73.9 |

^1^% Susceptible based on CLSI breakpoints. Cefepime-taniborbactam MICs were interpreted using a provisional susceptible breakpoint of ≤16 µg/mL (Karlowsky 2023). In the absence of CLSI breakpoints, meropenem-vaborbactam MICs against *P. aeruginosa* were interpreted using the EUCAST susceptible breakpoint of ≤8 µg/mL.

Supplementary Table 3 Composite, microbiologic, and clinical outcomes at Test of Cure in patients with select Enterobacterales species or *P. aeruginosa* at baseline, overall and in phenotypically and genotypically defined resistant subsets (extended microITT population)

| Organism/group; phenotypic^1^ or genotypic^2^ resistance subset (n; % of total) | Composite Success n/N (%) | |  | Microbiologic Success n/N (%) | |  | Clinical Success n/N (%) | |
| --- | --- | --- | --- | --- | --- | --- | --- | --- |
|  | Cefepime-taniborbactam | Meropenem |  | Cefepime-taniborbactam | Meropenem |  | Cefepime-taniborbactam | Meropenem |
| Enterobacterales overall (429; 100%) | 220/289 (76.1) | 86/140 (61.4) |  | 242/289 (83.7) | 97/140 (69.3) |  | 249/289 (86.2) | 113/140 (80.7) |
| *E. cloacae* complex (17; 4.0%) | 11/14 (78.6) | 1/3 (33.3) |  | 11/14 (78.6) | 1/3 (33.3) |  | 14/14 (100) | 3/3 (100) |
| Cefepime resistant (8; 47.1%) | 6/6 (100) | 1/2 (50.0) |  | 6/6 (100) | 1/2 (50.0) |  | 6/6 (100) | 2/2 (100) |
| Multidrug resistant^3^ (11; 64.7%) | 7/9 (77.8) | 1/2 (50.0) |  | 7/9 (77.8) | 1/2 (50.0) |  | 9/9 (100) | 2/2 (100) |
| ESBL^4^ (8; 47.1%) | 6/6 (100) | 1/2 (50.0) |  | 6/6 (100) | 1/2 (50.0) |  | 6/6 (100) | 2/2 (100) |
| AmpC^5^ (17; 100%) | 11/14 (78.6) | 1/3 (33.3) |  | 11/14 (78.6) | 1/3 (33.3) |  | 14/14 (100) | 3/3 (100) |
| *E. coli* (302; 70.4%) | 157/202 (77.7) | 62/100 (62.0) |  | 173/202 (85.6) | 71/100 (71.0) |  | 177/202 (87.6) | 80/100 (80.0) |
| Cefepime resistant (56; 18.5%) | 28/37 (75.7) | 9/19 (47.4) |  | 31/37 (83.8) | 11/19 (57.9) |  | 31/37 (83.8) | 15/19 (78.9) |
| Multidrug resistant^3^ (100; 33.1%) | 47/62 (75.8) | 25/38 (65.8) |  | 50/62 (80.6) | 29/38 (76.3) |  | 55/62 (88.7) | 31/38 (81.6) |
| ESBL^4^ (66; 21.9%) | 31/41 (75.6) | 13/25 (52.0) |  | 34/41 (82.9) | 16/25 (64.0) |  | 35/41 (85.4) | 19/25 (76.0) |
| *K. pneumoniae* (69; 16.1%) | 30/47 (63.8) | 14/22 (63.6) |  | 33/47 (70.2) | 16/22 (72.7) |  | 36/47 (76.6) | 16/22 (72.7) |
| Cefepime resistant (35; 50.7%) | 17/26 (65.4) | 7/9 (77.8) |  | 17/26 (65.4) | 7/9 (77.8) |  | 20/26 (76.9) | 7/9 (77.8) |
| Multidrug resistant^3^ (44; 63.8%) | 20/32 (62.5) | 9/12 (75.0) |  | 20/32 (62.5) | 10/12 (83.3) |  | 26/32 (81.3) | 9/12 (75.0) |
| Carbapenem resistant (9; 13.0%) | 6/7 (85.7) | 2/2 (100) |  | 6/7 (85.7) | 2/2 (100) |  | 7/7 (100) | 2/2 (100) |
| ESBL^4^ (36; 52.2%) | 16/26 (61.5) | 8/10 (80.0) |  | 16/26 (61.5) | 8/10 (80.0) |  | 20/26 (76.9) | 8/10 (80.0) |
| AmpC^5^ (3; 4.3%) | 1/2 (50.0) | 1/1 (100) |  | 1/2 (50.0) | 1/1 (100) |  | 2/2 (100) | 1/1 (100) |
| Carbapenemase (11; 15.9%) | 7/8 (87.5) | 3/3 (100) |  | 7/8 (87.5) | 3/3 (100) |  | 8/8 (100) | 3/3 (100) |
| *P. mirabilis* (20; 4.7%) | 9/10 (90.0) | 4/10 (40.0) |  | 10/10 (100) | 4/10 (40.0) |  | 9/10 (90.0) | 9/10 (90.0) |
| Cefepime resistant (4; 20.0%) | 2/2 (100) | 0/2 (0) |  | 2/2 (100) | 0/2 |  | 2/2 (100) | 2/2 (100) |
| Multidrug resistant^3^ (7; 35.0%) | 3/3 (100) | 1/4 (25.0) |  | 3/3 (100) | 1/4 (25.0) |  | 3/3 (100) | 4/4 (100) |
| ESBL^4^ (5; 25.0%) | 2/2 (100) | 0/3 (0) |  | 2/2 (100) | 0/3 (0) |  | 2/2 (100) | 2/3 (66.7) |
| *P. aeruginosa* overall (23; 100%) | 8/16 (50.0) | 4/7 (57.1) |  | 8/16 (50.0) | 5/7 (71.4) |  | 13/16 (81.3) | 6/7 (85.7) |
| Cefepime resistant (6; 26.1%) | 2/5 (40.0) | 1/1 (100) |  | 2/5 (40.0) | 1/1 (100) |  | 3/5 (60.0) | 1/1 (100) |
| Multidrug resistant^3^ (7; 30.4%) | 2/5 (40.0) | 1/2 (50.0) |  | 2/5 (40.0) | 1/2 (50.0) |  | 3/5 (60.0) | 2/2 (100) |
| Carbapenem resistant (5; 21.7%) | 1/3 (33.3) | 2/2 (100) |  | 1/3 (33.3) | 2/2 (100) |  | 2/3 (66.7) | 2/2 (100) |
| Carbapenemase (1; 4.3%) | 1/1 (100) | 0 |  | 1/1 (100) | 0 |  | 1/1 (100) | 0 |

^1^Phenotypes were assessed by reference broth microdilution (1, 2).

^2^β-lactamase genotypes among baseline isolates with MIC ≥2 µg/mL for aztreonam, ceftazidime, imipenem, and/or meropenem were determined by whole genome sequencing and in silico screening of β-lactamase genes.

^3^Resistance to ≥1 agent in ≥3 classes of antibacterial agents.

^4^Extended-spectrum β-lactamase genotype.

^5^Includes patients with pathogens carrying plasmidic *ampC* and pathogens at high risk of clinically significant AmpC production due to an inducible ampC gene (*E. cloacae* complex, *K. aerogenes*, and *C. freundii*; 3).

Supplementary Table 4 Summary of phenotypic and genotypic characteristics of serial *K. pneumoniae* isolates from Screening, Test of Cure, and Late Follow-up visits in Patient 3

|  | Visit (Study Day) | | |
| --- | --- | --- | --- |
|  | Screening | Test of Cure (20) | Late Follow-up (29) |
| MIC (µg/mL) (Interpretation) | | | |
| Cefepime | 64 (R) | >512 (R) | 512 (R) |
| Cefepime-taniborbactam^1^ | 1 (S) | 16 (S) | 2 (S) |
| Meropenem | 0.25 | >64 (R) | >64 (R) |
| Tobramycin | 0.5 (S) | 16 (R) | 0.5 (S) |
| Ampicillin-sulbactam | 128/64 (R) | >128/64 (R) | >128/64 (R) |
| Aztreonam | >64 (R) | >64 (R) | >64 (R) |
| Ceftazidime | >64 (R) | >64 (R) | >64 (R) |
| Ceftazidime-avibactam | 0.5 (S) | 8 (S) | 2 (S) |
| Imipenem | 0.12 (S) | >64 (R) | 64 (R) |
| Levofloxacin | >16 (R) | >16 (R) | >16 (R) |
| Meropenem-vaborbactam | 0.12 (S) | >64 (R) | 2 (S) |
| Piperacillin-tazobactam | >128 (R) | >128 (R) | >128 (R) |
| Trimethoprim-sulfamethoxazole | >64/1216 (R) | 64/1216 (R) | 64/1216 (R) |
| Sequence type | 258 | 258 | 258 |
| Single nucleotide polymorphisms (n) | (reference) | 3 | 4 |
| Insertion-deletion mutations (n) | (reference) | 5 | 4 |
| Plasmid (length [base pairs]); resistance gene content | | | |
| A (205,492); no β-lactamase genes identified | X | X | X |
| B (43,380); *bla*_SHV-12_ | X | X | X |
| C (113,639); *bla*_OXA-9_^2^, *bla*_KPC-2,_ *bla*_TEM-1_ |  | X | X |
| D (23,753); *bla*_KPC-2_, *aac*(6’)-*Ib* |  | X |  |

Abbreviations: S, Susceptible; R, Resistant (CLSI 2019).

^1^In the absence of breakpoints for cefepime-taniborbactam, MICs were interpreted using a provisional susceptible breakpoint of ≤16 µg/mL (4), for comparative purposes.

^2^*bla*_OXA-9_ had a premature stop codon and likely encoded a non-functional enzyme.

**Whole genome sequencing**

Total genomic DNA was used as input material for library construction. DNA libraries were prepared using the Nextera XT™ library construction protocol and index kit (Illumina, San

Diego, California, USA) and sequenced on a MiSeq Sequencer (Illumina) using a MiSeq

Reagent Kit v3 (600 cycle). Sequencing reactions were initially performed to achieve short DNA

read lengths of up to 300-bp and an average genome coverage depth of approximately 30×.

FASTQ format files for each sample set were assembled independently using de novo assembler

SPAdes 3.11.1 and a FASTA-format file of contiguous sequences was obtained. These

contiguous sequences were submitted to quality assurance procedures according to established

SOPs. If any quality control (QC) parameters were not met, the isolate was re-sequenced.

**Other microbiologic outcomes**

There were no patients with superinfection, defined as isolation of a pathogen of a species other than that of the causative baseline pathogen at ≥10^5^ CFU/mL in a urine culture obtained during study drug therapy, in a patient with new or worsening signs and symptoms of infection requiring alternative antimicrobial therapy. Two patients had a new infection, defined similarly to superinfection but occurring after completion of study drug therapy. One of these patients (meropenem group) had *E. coli* in urine at baseline, *Enterococcus faecium* in urine at EOT, was a composite success at EOT, and was a composite non-responder at both TOC and LFU owing to clinical failure at each of these two visits. The other patient (cefepime-taniborbactam group) had *Klebsiella variicola* in urine at baseline, *C. freundii* complex at TOC and LFU, and was a composite non-responder at EOT, TOC, and LFU owing to clinical failure despite microbiologic success at each of these three visits.

**Evaluation of post-baseline isolates with apparent increases in cefepime-taniborbactam and meropenem MIC**

Post-baseline isolates with a ≥4-fold greater MIC to study drug received in that patient compared to the MIC against the corresponding baseline isolate of the same species were recovered from three patients in the extended microITT population. Of these patients, two were in the cefepime-taniborbactam group (one patient with *E. coli*; one patient with *K. pneumoniae*) and one was in the meropenem group (*K. aerogenes*).

In the following two cases, the post-baseline strains had MICs that were several doubling dilutions below the provisional susceptible breakpoint for cefepime-taniborbactam, or the susceptible breakpoint for meropenem.

Patient 1 (cefepime-taniborbactam group) had *E. coli* recovered in urine at baseline, TOC, and LFU. The cefepime-taniborbactam MICs were 0.03 μg/mL at baseline, 0.06 μg/mL at TOC, and 0.12 μg/mL at LFU. The isolates at TOC and LFU (both ST162) were clonally different than the baseline isolate (sequence type [ST] 69).

Patient 2 (meropenem group) had *K. aerogenes* recovered at baseline, TOC, and LFU. The meropenem MICs were 0.06 μg/mL at baseline, 0.25 μg/mL at TOC, and 0.06 μg/mL at LFU. All three isolates had the same clonal type (ST190).

Patient 3 (cefepime-taniborbactam group) had *K. pneumoniae* recovered at baseline, TOC, and LFU with strikingly different phenotypic and genotypic profiles (Supplementary Table 4). All isolates were of ST258 and were therefore clonally related. However, when the genome sequences of the TOC and LFU strains were compared to that of the baseline strain, the TOC isolate had 3 single nucleotide polymorphisms (SNPs) and 5 insertion/deletion mutations (indels), whereas the LFU strain had 4 SNPs and 4 indels. In addition, the TOC and LFU isolates had an additional plasmid (denoted as plasmid “C” in Supplementary Table 4) containing *bla*_OXA-9_ (truncated), *bla*_KPC-2_, and *bla*_TEM-1_ genes that was not detected in the baseline isolate, explaining the higher MICs for β-lactams including cefepime, imipenem, and meropenem in the TOC and LFU isolates compared to the baseline isolate.

Furthermore, the TOC isolate in this patient had an additional plasmid (denoted as plasmid “D” in Supplementary Table 4) that was not present in either the baseline or LFU strains. This unique plasmid also carried *bla*_KPC-2_, as well as *aac*(6’)-*Ib* encoding an aminoglycoside 6'-N-acetyltransferase. The difference in cefepime-taniborbactam MIC between baseline (1 μg/mL), TOC (16 μg/mL) and LFU (2 μg/mL) strains (and the corresponding differences in meropenem-vaborbactam MIC [0.12, >64, and 2 µg/mL] and ceftazidime-avibactam MIC [0.5, 8, and 2 µg/mL] between these strains) may be attributed to this additional copy of *bla*_KPC-2_ in the TOC strain, against which MICs of all β-lactams were also higher than those against the baseline and LFU strains. Concordantly, the increased tobramycin MIC (16 μg/mL) against the TOC strain may be attributed to the *aac*(6’)-*Ib* element that was absent from baseline and LFU strains (each of which had a tobramycin MIC of 0.5 μg/mL).

Based on previous studies of other sequentially isolated strains (5, 6, 7), it is likely that the TOC and LFU strains in Patient 3 did not originate from the baseline strain due to (i) the frequency of SNPs and indels within the short timeframe (20 and 29 days), and (ii) the additional plasmid(s) compared to the baseline strain as summarized above and in Supplementary Table 4. Furthermore, the TOC and LFU strains were genetically closer to each other than either strain was to the baseline counterpart.

The strains in Patient 3 belong to a prevalent and international high-risk clonal type (and common sequence type, ST258) that has established itself in nosocomial environments and causes infections in patients worldwide. Patient 3 therefore likely had separate infection episodes, one initially due to the baseline strain and one or more subsequent episode(s) due to the TOC and/or LFU strains. Further analyses that were beyond the scope of this Phase 3 registrational study would have been required to understand whether this patient was colonized with these strains in another anatomical compartment such as the gut, vaginal canal, or perineum, or had another potential reservoir for persistent/recurrent infection such as intracellularly localized pathogens in the bladder epithelium.

**REFERENCES**

1. CLSI Standard M07, 11th Edition, "Methods for Dilution Antimicrobial Susceptibility Tests for Bacteria That Grow Aerobically, 11th Edition", Clinical and Laboratory Standards Institute (CLSI), Wayne, PA, 2018.

2. CLSI Supplement M100, 29th Edition, "Performance Standards for Antimicrobial Susceptibility Testing, 29th Edition. ", Clinical and Laboratory Standards INstitute, Wayne, PA, 2019.

3. Tamma PD, Aitken SL, Bonomo RA, Mathers AJ, van Duin D, Clancy CJ. Infectious Diseases Society of America 2023 Guidance on the Treatment of Antimicrobial Resistant Gram-Negative Infections. *Clin Infect Dis*. July 2023. doi:10.1093/cid/ciad428

4. Karlowsky JA, Hackel MA, Wise MG, et al. In Vitro Activity of Cefepime-Taniborbactam and Comparators against Clinical Isolates of Gram-Negative Bacilli from 2018 to 2020: Results from the Global Evaluation of Antimicrobial Resistance via Surveillance (GEARS) Program. *Antimicrob Agents Chemother*. December 2022. doi:10.1128/aac.01281-22

5. Cheng S, Fleres G, Chen L, et al. Within-Host Genotypic and Phenotypic Diversity of Contemporaneous Carbapenem-Resistant Klebsiella pneumoniae from Blood Cultures of Patients with Bacteremia. *MBio*. November 2022. doi:10.1128/mbio.02906-22

6. Mulvey MR, Haraoui L-P, Longtin Y. Multiple Variants of Klebsiella pneumoniae Producing Carbapenemase in One Patient. *N Engl J Med*. 2016;375(24):2408-2410. doi:10.1056/NEJMc1511360

7. Wylie KM, Wylie TN, Minx PJ, Rosen DA. Whole-Genome Sequencing of Klebsiella pneumoniae Isolates to Track Strain Progression in a Single Patient With Recurrent Urinary Tract Infection. *Front Cell Infect Microbiol*. 2019;9. doi:10.3389/fcimb.2019.00014
